# Estimating the Carbon Emission of Construction Waste Recycling Using Grey Model and Life Cycle Assessment: A Case Study of Shanghai

**DOI:** 10.3390/ijerph19148507

**Published:** 2022-07-12

**Authors:** Ting Wang, Kaiyi Li, Defu Liu, Yang Yang, Dong Wu

**Affiliations:** 1 College of Urban Construction and Safety Engineering, Shanghai Institute of Technology, Shanghai 201418, China; pauline_wt@163.com; 2 Key Laboratory of Environmental Pollution Monitoring and Disease Control, Ministry of Education, Guizhou Medical University, Guizhou 550001, China; 52203903025@stu.ecnu.edu.cn; 3 Shanghai Key Laboratory for Urban Ecological Processes and Eco-Restoration, Shanghai Engineering Research Center of Biotransformation of Organic Solid Waste, School of Ecological and Environmental Sciences, East China Normal University, Shanghai 200241, China; 4 Shanghai Construction No. 4 (Group) Co., Ltd., Shanghai 201103, China; ldflovezm@163.com; 5 Department of Building and Real Estate, The Hong Kong Polytechnic University, Hong Kong SAR, China; yyang@polyu.edu.hk

**Keywords:** construction waste management, generation amount, resource recycling, emission abatement, China

## Abstract

Great efforts have been exerted in reducing carbon emissions in design, construction and operation stages. However, little attention is paid to the quantification of carbon emissions in construction waste recycling at the end-of-life stage. This study aims to quantitatively analyze the carbon emission of construction waste in Shanghai City, PR China. A grey model is used to forecast the generation amount of construction waste, and a life cycle assessment is performed to estimate the carbon emission of construction waste. In this study, both the carbon emission of recycling activities (environmental costs), and the equivalent amount of carbon generated from alternative materials (environmental benefit) are considered. Here, recycling 1 ton (t) of construction waste in Shanghai can save 100.4 kg CO_2_−e. The total carbon-emission-saving potential can be increased from 0.31 million t CO_2_−e (2022) to 0.35 million t CO_2_−e (2031). The carbon emission of recycling concrete, brick, steel, wood and mortar, identified as the key components of construction waste, is investigated. This research can help to reduce carbon emissions and further achieve carbon neutrality for Shanghai City. The proposed methods can also be applied to other regions, especially when the data for construction waste are insufficient.

## 1. Introduction

Global warming strongly challenges and jeopardizes the sustainable development of the environment and human society. Countries worldwide make joint efforts to reduce carbon emissions [1], which is viewed as the most important contributor to global warming [2]. As the largest developing country and the largest carbon emitter in the world, China has committed to achieve carbon neutrality by 2060, in which the 60–65% of industrial carbon emission intensity should be reduced in comparison to the 2005 level [3].

The building sector reportedly contributes to 40% of the total carbon emissions worldwide [4]. With the burgeon of the building industry market in China, a large amount of construction and demolition (C&D) waste is generated annually. China is the largest producer of C&D waste in the world [5]. In accordance with existing studies, the annual generation amount of C&D waste in the US is around 485 million t, 870 million t in Europe [6], and up to 1500 million t in China [7]. Huge quantities of C&D waste will lead to negative environmental impacts, such as loss of habitat when land is used as new landfills [8]. Thus, many regulations and strategies have been implemented to reduce, reuse and recycle C&D waste in the past few decades. However, the status quo of C&D waste management in China is unsatisfactory, and the recycling rate is lower than that of some developed economies. Currently, most of C&D wastage is still transported to landfills for disposal [9]. One of the most important reasons for this low-recycling dilemma is the lack of reliable data sources due to the rudimentary statistical systems in these developing regions [1,9]. At present, many studies concentrated on how to reduce the carbon emission of buildings during the design [10], construction and operation stages [11], whereas minimal attention has been paid to the quantifying the CO_2_ emission of C&D recycling at the end-of-life stage.

Therefore, this research aims to forecast the generation amount of construction waste and estimate the carbon emission of construction waste recycling. Shanghai City, PR China, is used as a case study. Subsequent to this introduction is the literature review in Section 2 to review the literature on the environmental impact of construction waste and recycling practices of each waste composition. Section 3 discusses the two main methods, namely the grey model (GM 1.1) and life cycle assessment (LCA), adopted in this research. Section 4 presents the results, including the results of construction waste generation, and the results of carbon emission of construction waste recycling. Section 5 provides the conclusion.

## 2. Literature Review

### 2.1. Environmental Impact of Construction Waste

C&D waste is generally defined as the solid waste generated from construction, demolition and renovation activities, and can occasionally be called construction waste. It usually comprises inert (e.g., concrete and brick) and noninert materials (e.g., wood and plastic) [12]. Shanghai is a megacity in eastern China where 25 million people live. In such a highly populous city, the generation rate of C&D waste can be astonishing. In accordance with the study of Jin et al. [13], a 1 km^2^ land area can generate about 22,713 t of C&D waste in Shanghai. With the rapid development of urbanization, the amount of C&D waste is expected to increase in the future. Ding et al. [9] quantified the components of building-related C&D waste in Shanghai, and the main compositions of construction waste include brick or block (38.3%), mortar (1.1%), steel (6.5%), wood (11.2%) and concrete (42.9%). Comparatively, demolition waste contains brick or block (63.8%), mortar (2.1%), steel (3.1%), wood (8.4%) and concrete (22.6%). New construction waste must be separated from demolition waste because the composition and unit generation amount of demolition waste differ remarkably from new construction waste [9,14]. In this study, we only evaluated the solid waste generated from new construction activities in Shanghai (excluding construction clay and mud).

As effective waste management strategies, the reduction, reuse and recycling of construction waste have received much attention worldwide. Many strategies and policies have been utilized to effectively reduce construction waste. For example, transportation infrastructures are encouraged to use recycled aggregates, and the replacement rate of recycled materials should be greater than 30% in Shanghai [15]. However, the recycling rate of construction waste in mainland China is lower than the developed regions [16]. The recycling rates of C&D waste reached up to 70–80% in some European countries, whereas the recycling rate in developing areas, such as China, is lower than 50% [17]. Some studies were conducted on the environmental impact of construction waste due to its considerable high generation rate and detrimental impacts on the environment. Su et al. [18] adopted a mixed method of building information modelling, a geographic information system and LCA to develop an evaluation model for building-related demolition waste and to assess its environmental impact. Liu et al. [19] used system dynamics to estimate the environmental impact of C&D waste in Guangzhou, PR China. One of the most remarkable environmental impacts of construction waste disposal is carbon emissions. However, some studies only concentrate on quantifying the carbon emission of construction waste. Peng et al. [1] quantified the embodied carbon mitigation potentials of recycling C&D waste generated in the Greater Bay Area, China. Wu et al. [17] utilized a streamlined LCA to evaluate the carbon emission of construction waste in Shenzhen City.

### 2.2. Recycling Practices of Construction Waste

**Figure 1:**
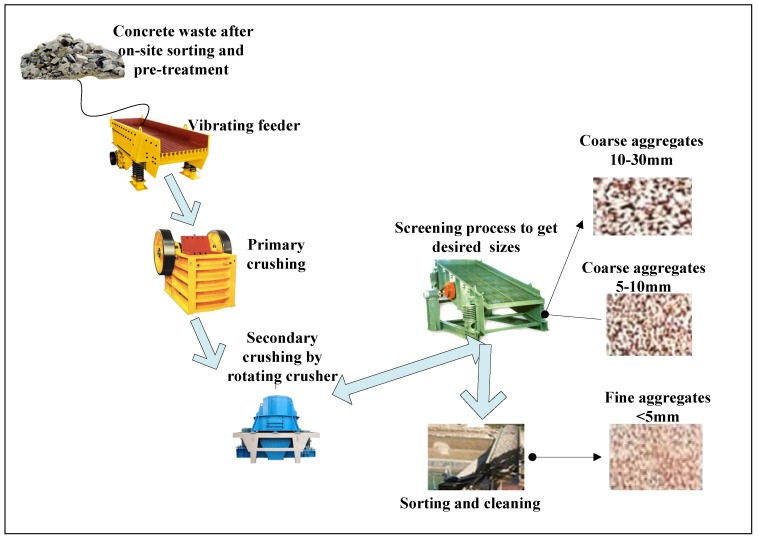
A simplified processing flow of the recycling concrete waste (Source: investigated by the authors in Qibao, Minhang district, Shanghai).

When construction waste is generated on-site, manual separation can be firstly used to separate recoverable materials before transportation to landfills or recycling plants [20]. The recycling rate of concrete waste in Shanghai is approximately 100% because its composition is simple and easy to recycle [16]. Recycling treatment of concrete waste usually involves crushers, screeners, magnetic separators, wind sifting, and manual separation. In this study, we targeted the most commonly used recycling treatments of concrete waste, namely reproducing the aggregates from crushed concrete. As shown in Figure 1, a simplified process of concrete recycling treatment in Shanghai usually includes primary and secondary crushing to produce aggregates with various size fractions [21]. The recycled aggregates are regulated to be compliant with the requirements concerning particle density and fine particles, being in line with the Technical Code for Application of Recycled Aggregated Concrete in China [2]. The qualified recycled aggregates can be used in concrete, ready-mixed mortar or subgrade [16].

**Figure 2:**
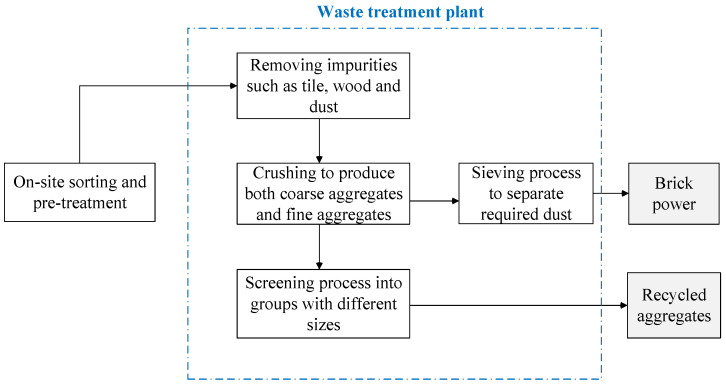
A simplified map for recycling brick waste (Source: Wang et al. [2]).

Brick waste is often contaminated by mortar and plaster. As such, on-site sorting is firstly performed before transportation to a recycling plant. As reported by Tam and Tam [22], in Hong Kong, the most common practice of brick waste recycling is crushing to make filling materials and hard core. Similar to concrete recycling, the treatment process of brick waste recycling involves crushing, screening and grouping in accordance with different sizes [23]. Figure 2 shows a simplified process of brick recycling that produces aggregates and brick power. The recycled production needs to meet the requirements, such as comprehensive strength and flexural strength [24], before cement is replaced with mortar.

Ferrous metal, such as steel, often accounts for an extremely small proportion of construction waste but usually has high recycling rates due to its magnetic substances with high market value [25]. Reinforcing bars from concrete waste can be recycled for remelting. The treatment process of steel often involves charging, melting and decarburization [26]. Similarly, wood waste accounts for a small amount of construction waste, and is collected by scrap dealers and can be incinerated as fuel after on-site sorting [17]. Regarding the mortar waste, separation from the recycling process of concrete is not conducted. However, the value of cement mortar is relatively low when compared with other waste compositions [15]. This is because mortar waste is built with high porosity and water absorption rates that can weaken the strength and mechanical performance of recycled aggregates made by concrete waste [27,28]. Thus, we assume that the treatment process of recycling mortar waste is the same as that of concrete, and the value of recycled mortar is ignored.

### 2.3. Shanghai City, China

Shanghai is a megacity located in in eastern China. It is one of the four direct-administered municipalities of the PR, China. With a population of 24.28 million, it is the most populous urban area in China and the third most populous city in the world. Additionally, the port of Shanghai is the world’s busiest container port.

This research focuses on Shanghai city due to three reasons. Firstly, every carbon quantification study needs to be contextualized in specific areas in order to obtain the data [1]. Secondly, as a global center for finance, technology, manufacturing and transportation, Shanghai is one of the most important economic regions in China. Due to the rapid development of the construction industry during the past few decades, this city is facing a dilemma of reducing C&D waste generation, while improving the reusing, and recycling of such waste. Thirdly, the local government prioritizes sustainable development in their reports and also carries out action plans, aiming at promoting the recycling of C&D waste and reducing CO_2_ emissions in the construction industry [15].

## 3. Research Methods

**Figure 3:**
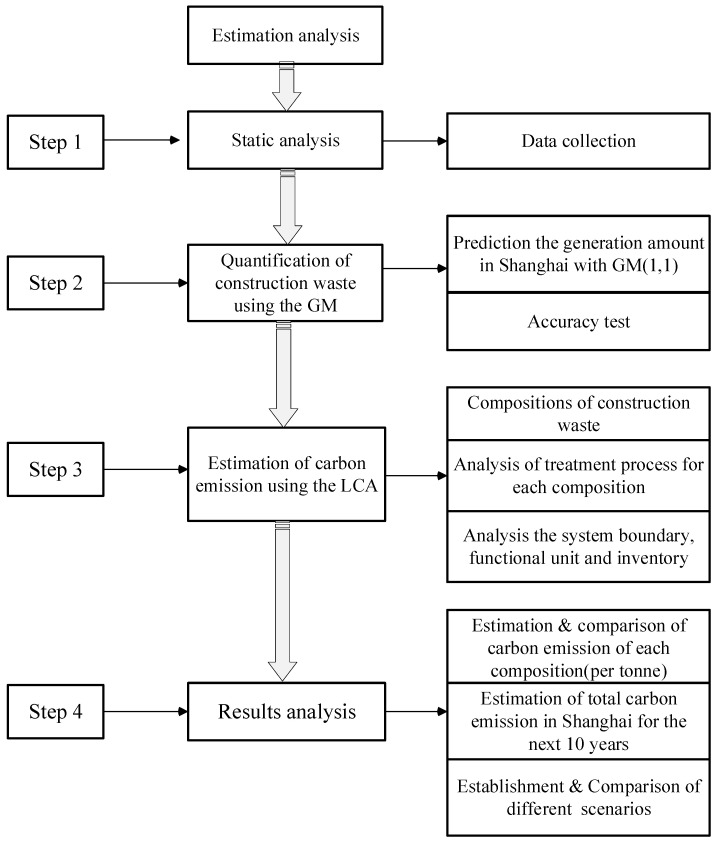
Research framework of this study (LCA: life cycle assessment; GM: grey model).

This study firstly adopts the grey model to forecast the generation amount of construction waste in Shanghai. LCA is then used to estimate their carbon emissions. The method framework of the present study is illustrated in Figure 3.

### 3.1. Grey Model (1,1)

The grey model, which is constructed from the grey system theory, focuses on the insufficient information available, or the uncertainty of information that is [29]. It has been extensively utilized in the industries of finance, economics and the quantification of construction waste generation [30]. A major advantage of this method is that it can help predict problems with less data [29], which is extremely suitable for the current study. As the core of grey theory, the grey model (1,1) is a first-order linear differential equation of a single variable selected to forecast the generation amount of construction waste in Shanghai City in the next 10 years.

Step I: Set $x^{\left(0\right)}$ as a non-negative sequence:

(1) $$x^{\left(0\right)}=(x^{\left(0\right)}(1),x^{\left(0\right)}(2),\cdots x^{\left(0\right)}(n))$$

Accumulate the original data of $x^{\left(0\right)}$ to weaken the volatility randomness of random sequences, thereby obtaining $\;x^{\left(1\right)}$.

(2) $$x^{\left(1\right)}=(x^{\left(1\right)}(1),x^{\left(1\right)}(2),\cdots x^{\left(1\right)}(n)),x^{\left(1\right)}(k)=\sum_{i=1}^{k}x^{\left(0\right)}(i);k=i,2\cdots n$$

Step II: Generate the equal weight sequence of the adjacent mean of $\;x^{\left(1\right)}$, which is called $z^{\left(1\right)}$.

(3) $$z^{\left(1\right)}(k)=0.5x^{\left(1\right)}(k-1)+0.5x^{\left(1\right)}(k),k=2,3\cdots n$$

Step III: Using the least squares method yields:

(4) $$\overset{\wedge }{a}={\left(B^{T}B\right)}^{-1}B^{T}Y_{n}$$

where:

(5) $$B=\left[\begin{array}{cc} -z^{\left(1\right)}(2) & 1\\ -z^{\left(1\right)}(3) & 1\\ \vdots & \vdots \\ -z^{\left(1\right)}(n) & 1 \end{array}\right]=\left[\begin{array}{cc} -\frac{1}{2}(x^{\left(1\right)}(1)+x^{\left(1\right)}(2)) & 1\\ -\frac{1}{2}(x^{\left(1\right)}(2)+x^{\left(1\right)}(3)) & 1\\ \vdots & \vdots \\ -\frac{1}{2}(x^{\left(1\right)}(n-1)+x^{\left(1\right)}(n)) & 1 \end{array}\right],\;Y_{n}=\left[\begin{array}{c} x^{\left(0\right)}(2)\\ x^{\left(0\right)}(3)\\ \vdots \\ x^{\left(0\right)}(n) \end{array}\right]$$

where B is a data matrix.

Step IV: Establish a one-order differential sequence of time *t* for $x^{\left(1\right)}$,

(6) $$\frac{dx^{\left(1\right)}}{dt}+ax^{\left(1\right)}=u$$

The discrete time response function can be represented as follows:

(7) $${\overset{\wedge }{x}}^{\left(1\right)}(t+1)=(x^{\left(1\right)}(1)-\frac{u}{a})e^{-at}+\frac{u}{a}$$

We can conduct residual analysis on the prediction results by using the following equation:

(8) $$\begin{array}{l} {\overset{\wedge }{x}}^{\left(1\right)}(k)=(x^{\left(0\right)}(1)-u/a)e^{-a(k-1)}+u/a \end{array}$$

### 3.2. LCA

**Figure 4:**
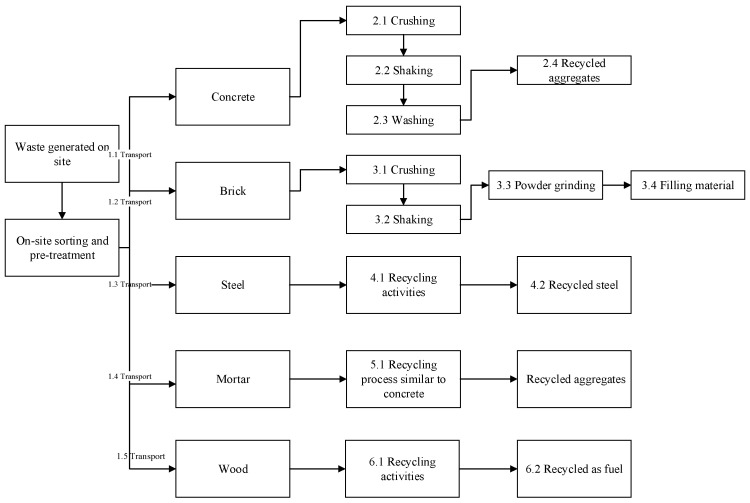
The system boundary of recycling practice of construction waste (Source: Wang et al. [2]).

LCA is widely used to evaluate the carbon emission of C&D waste, such as the studies conducted by Wang et al. [2] and Peng et al. [1]. The system boundary is selected based on the aims of this study. As illustrated in Figure 4, the boundary encompasses the transport of construction waste to waste treatment in the recycling plants. The carbon emission of internal transportation is ignored because the transportation distance in the recycling plant is extremely short. The functional unit is 1 t of construction waste in this study. Table 1 represents the composition of construction waste in Shanghai, which is based on Ding and Xiao [9]. Other assumptions are made to calculate the input carbon emissions. 

## 4. Results and Analysis

### 4.1. Results of Construction Waste Generation

#### 4.1.1. Data Collection and Calculation

This study selected the approach shown in Equation (9) to estimate the annual generation amount of construction waste. This method is based on some measurements of construction activity levels in a region (by area, m^2^) and the average waste generation per construction area (t/m^2^) to quantify the generation amount of construction waste [9]. In this study, the annual data of the construction area in Shanghai (CA) are obtained from Shanghai Statistics Yearbook 2021, which is issued by Shanghai Statistics Bureau (http://tjj.sh.gov.cn/). $G_{c}$ is 0.04 t/m^2^, and is cited from the Handbook of Green Building Evaluation Standards (GB/T 50378—2019) enacted by the Ministry of Housing and Urban–Rural Development of China. The data of construction area and construction waste from 2010 to 2021 are presented in Table 2.

(9) $$W=CA*G_{c},\;$$

where CA represents the construction area, and $G_{c}$ is the average generation amount of construction waste per construction area.

In accordance with Equations (1)–(8), we can calculate the sequence of *B* and $Y_{n}$, as shown in Table 3.

#### 4.1.2. Model Verification and Result Analysis

**Figure 5:**
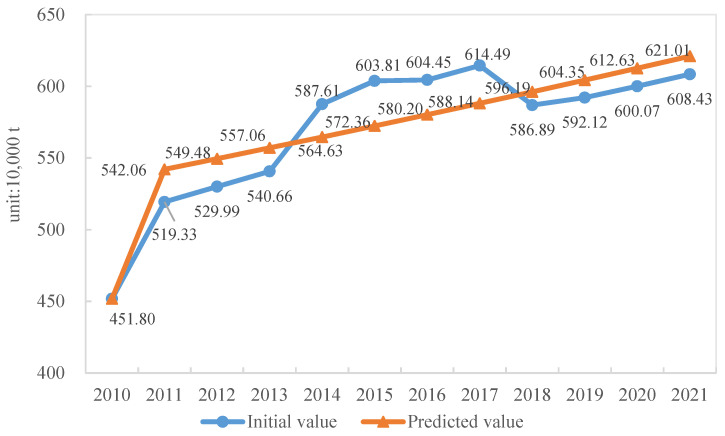
Comparison of initial and forecasting amount of construction waste.

In the GM model, the value of the mean square error ratio is 0.32, which is lower than 0.35, indicating that the model accuracy meets the requirement [32]. The value of small error probability *P* is 1, which is higher than 0.95, presenting that the model accuracy is excellent [33]. As shown in Figure 5, the maximum relative error corresponding to the predicted value is 3.9%. In accordance with the grey system theory, the acceptable error ranges from 0 to 0.2 [33]. Thus, the prediction accuracy meets the requirement.

**Figure 6:**
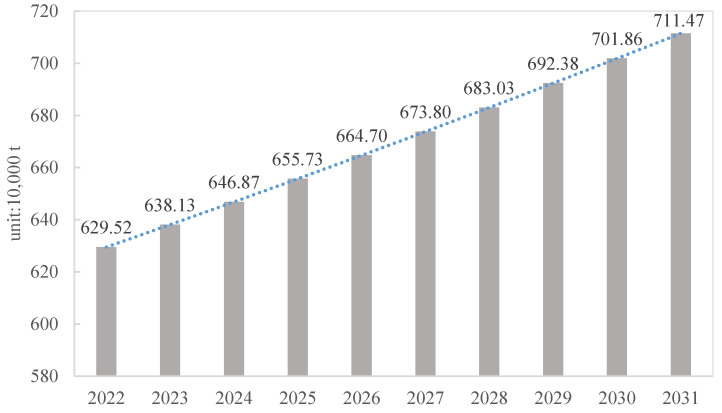
Forecasting generation amount of construction waste in Shanghai from 2022 to 2031.

The predicted generation amount of construction waste in Shanghai from 2022 to 2031 is shown in Figure 6, with values of 629.52, 638.13, 646.87, 655.73, 664.70, 673.80, 692.38, 701.86, 701.86 and 711.47 (unit 10,000 t), respectively. The annual generation amount of construction waste will continuously increase in the next 10 years but at a slower rate.

### 4.2. Results of the Carbon Emission of Construction Waste Recycling

#### 4.2.1. Data Collection and Calculation

- Transportation

The mean distance of waste transportation from a construction site to a waste treatment plant is obtained by using the following equation:

(10) $$D=L_{i}*S_{i}$$

where *D* represents the average transportation distance, and *i* refers to a distance in Shanghai. $S_{i}$ is specific to the percentage of district *i*’s construction projects in Shanghai, and $L_{i}$. is the average transportation distance in district *i*. In accordance with the public information issued on the Shanghai Construction Muck Comprehensive Service Monitoring Platform, the total number of current construction sites, their district distributions and construction waste treatment plants can be obtained. Ten districts and six construction waste treatment plants were considered. We assumed that the construction waste is transported from the site to the nearest treatment plants, and the overall average transportation distance is calculated as 35.29 km (Table 4). Steel recycling and wood recycling plants are located in Shanghai’s rural areas. Thus, the transportation distance for the two is assumed to be 50 km [2].

- Carbon emissions of key activities

Table 5 shows the carbon emissions of key activities in this study. The direct carbon emissions of concrete, brick and mortar recycling activities and the indirect emissions of diesel and electricity production were considered. This method is in agreement with the international standards and previous studies, such as that of Wang et al. [2], which states that the emissions regarding electricity use should also consider the generation of electricity. Considering the large amount of data required in LCA studies, data uncertainty becomes an important problem. This study mainly uses the data from China, such as Gu [23] and Yang [34], to mitigate data uncertainty.

#### 4.2.2. Result Analysis

- Carbon emission of each waste composition (1 t)

The carbon emissions of the recycling activities in terms of concrete, brick, steel, mortar and wood are listed in Table 6. In this study, recycling credits represent the equivalent amount of carbon generated from alternative materials. Additionally, they are expressed as negative values in this table, indicating their environmental benefits. Here, environmental benefits were obtained from the steel and wood recycling. However, concrete, brick and mortar recycling imposed notably strong negative environmental impacts. Interestingly, as shown in Table 5, the carbon emission of each type of construction waste varied substantially. For example, the recycling of steel leads to the generation of the highest credits at 1811.09 kg CO_2_−e per capita (1 t). This is because steel recycling heavily reduces the CO_2_ generated from the production process by approximately 30% [37]. Similarly, wood recycling leads to an environmental credit with a carbon emission of 1.24 kg CO_2_−e because wood as a raw material can be obtained from nature, and its production generates less CO_2_ [17]. Comparatively, recycling 1 t of brick generates the most significant amount, at 35.82 kg CO_2_−e. Assumptions concerning the usage of recycled materials could also alter the carbon emission. In this study, bricks are reused and recycled as filling materials. Therefore, the benefits derived from the recycling as filling materials may be less than the direct use of recycled bricks. As shown in Table 6, the recycling credits of mortar are ignored because recycled mortar is normally not reused in construction activities in Shanghai [15]. Recycling 1 t of concrete generates 8.43 kg CO_2_−e, indicating the lowest negative impact amongst brick, mortar and concrete recycling. These findings are in accordance with previous studies, such as that of Wang et al. [2] and Mercante, I.T. et al. [38].

- Carbon emission of recycling 1 t of construction waste in Shanghai

**Figure 7:**
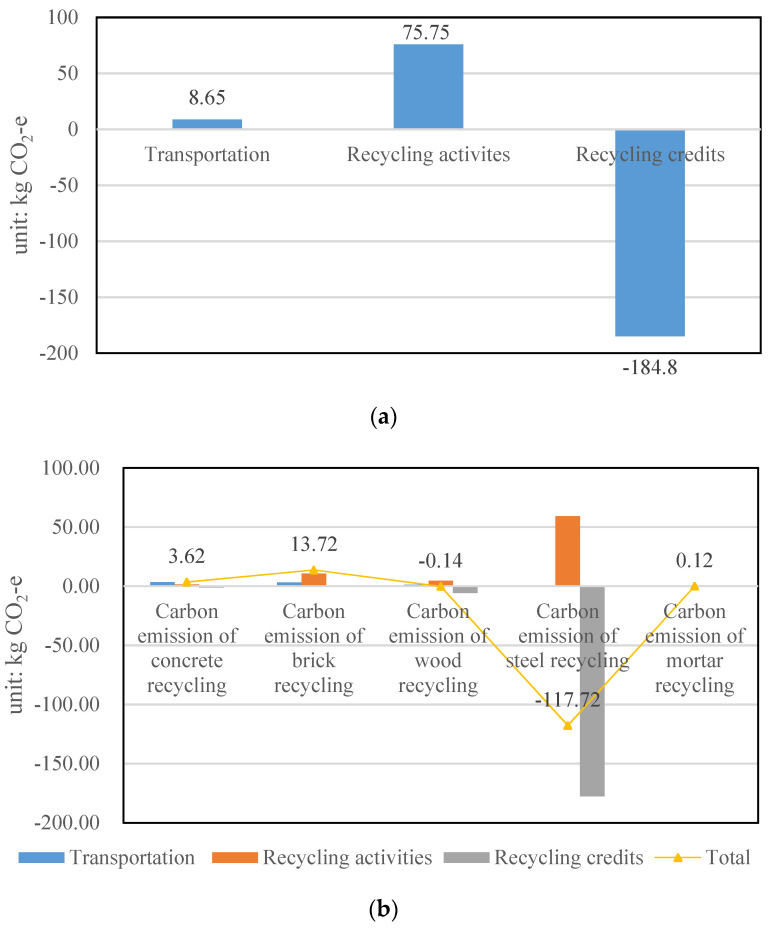
(**a**) The carbon emission of recycling 1 t of construction waste in Shanghai (by activities); (**b**) The carbon emission of recycling 1 t of construction waste in Shanghai (by waste composition).

As shown in Figure 7a, recycling 1 t of construction waste in Shanghai can save 100.4 kg CO_2_−e. The transportation stage generates a total of 8.65 kg CO_2_−e, and recycling activities lead to a total amount of 75.75 kg CO_2_−e. By contrast, recycling credits gained from recycled waste products save 184.80 kg CO_2_−e. As shown in Figure 7b, concrete recycling, brick recycling and mortar recycling generate 3.62, 13.72 and 0.12 kg CO_2_−e, respectively. Comparatively, steel recycling and wood recycling lead to a carbon emission reduction of 117.72 kg CO_2_−e and 0.14 kg CO_2_−e. As illustrated in Figure 7b, concrete recycling contributes the most significant emission reduction in the transportation stage, with a value of 3.45 kg CO_2_−e, whereas mortar recycling is the lowest with 0.09 kg CO_2_−e. Steel recycling produces the largest amount of carbon emission reduction in recycling activities (59.23 kg CO_2_−e), followed by brick recycling (10.69 kg CO_2_−e), wood recycling (4.47 kg CO_2_−e), concrete recycling (1.33 kg CO_2_−e) and mortar recycling (0.03 kg CO_2_−e). For recycling credits, the five waste compositions differ significantly, and steel recycling exhibits the most significant emission reduction of 177.70 kg CO_2_−e which is in line with previous studies, such as that conducted by Sandulescu, E. [39].

- Sensitivity analysis

One of the key issues of the LCA approach is uncertainty. Methods to quantify uncertainty usually include random sampling, second-order probability methods, Bayesian methods, and sensitivity analysis [40]. In this study, calculated outcomes are based on some assumptions which are not often quantified using techniques such as the Monte Carlo analysis and Bayesian methods. Sensitivity analysis is an uncertainty analysis method that explores the degree of influence on the results when certain parameters change, and it is often adopted in LCA studies to analyze uncertainties [17]. Thus, the sensitivity analysis is used in this study and two key sensitivity factors, including transportation distance and recycling rate, are selected. In sensitivity analysis, the variation range of the two sensitivity factors is set from −10% to 10% of the original value, and Table 7 shows the extent to which carbon emissions change as the sensitivity factor changes. As represented in Table 7, for example, when the transportation distance increases by 10%, then the carbon emission of concrete recycling would enhance 9.55%. Additionally, changes in transportation distance have the greatest impact on the carbon emission of concreter recycling, while changes in recycling rate pose the most obvious impact on carbon emission of brick recycling. Additionally, all rates of change are in the range −10% to 10%, which is within an acceptable uncertainty range [40].

- Scenario analysis: Carbon emission of construction waste recycling in Shanghai from 2022 to 2031

**Figure 8:**
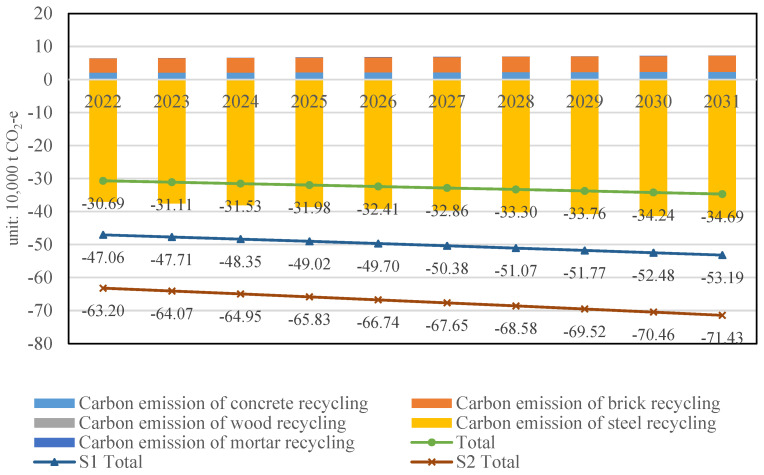
The carbon emission of construction waste recycling in Shanghai from 2022 to 2031.

The estimated carbon emission of construction waste recycling in Shanghai from 2022 to 2031 is illustrated in Figure 8. The histograms only give the data under the current scenario, where the recycling rates of concrete waste, brick waste, wood waste, steel waste, and mortar waste are 90%, 50%, 50%, 50% and 40%, respectively. As shown in Figure 8, the carbon-emission-saving potential is increased from 2022 (0.31 million t CO_2_−e) to 2031 (0.35 million t CO_2_−e). The carbon emission of mortar waste recycling accounts for the smallest proportion of all compositions because the recycling credits of mortar are ignored in this study. The carbon emission of steel waste recycling accounts for the largest proportion because steel recycling can help reduce the CO_2_ generated from the production process by approximately 30% [36].

In this study, two scenarios are set up to estimate the carbon emission of construction waste recycling in Shanghai under different recycling rates. In accordance with governmental planning, the recycling rate of construction waste in Shanghai will increase to 75% by 2025 [13]. Thus, in the scenario 1 (S1), the recycling rate of concrete waste, brick waste, wood waste, steel waste and mortar waste are set as 90%, 75%, 75%, 75% and 75%, respectively. In the scenario 2 (S2), the recycling rates are set as 100%, which can be regarded as an ideal scenario. As shown in Figure 8, three lines (current scenario, S1 and S2) represent the total amount of carbon emissions of construction waste recycling in Shanghai for the next decade. With the improvement of the recycling rate of construction waste, construction waste recycling can lead to more environmental benefits and can help to prevent more carbon emissions. The predictions show that approximately 0.35 million t of carbon emissions will be saved in the year 2031 compared with the current scenario. The environmental benefits of construction waste recycling in 2031 under S1 and S2 are 0.53 million and 0.71 million t CO_2_−e, respectively. Compared with the current scenario, the carbon emission reduction under S1 and S2 will be 51.43% and 102.86%. These study outcomes, in which the recycling of construction waste leads to important reduction in CO_2_ emission, echo earlier research in other regions, such as that conducted by Islam, R. et al. in Bangladesh [41] and Taffe, W.Z. et al. in Ethiopia [42].

## 5. Conclusions

The rapid development of urbanization has led to significant construction activities and the generation of a huge amount of construction waste. Waste recycling can significantly reduce carbon emissions and help promote sustainable development. This study forecasted the generation amount of construction waste and the carbon emission of construction waste recycling on the basis of GM (1.1) and LCA by using Shanghai City as a case study. The results show that recycling 1 t of construction waste in Shanghai can save 100.4 kg carbon emission, where concrete recycling, brick recycling and mortar recycling generate 3.62, 13.72, and 0.12 kg CO_2_−e, and steel recycling and wood recycling lead to a carbon emission reduction of 117.72 and 0.14 kg CO_2_−e. The total amount of construction waste generated is estimated to be around 0.63 million and 0.77 million t in 2022 and 2031, respectively. If they are recycled in accordance with current recycling rate in Shanghai, the total carbon-emission-saving potential will increase from 0.31 million t CO_2_−e (2022) to 0.35 million t CO_2_−e (2031). Two different recycling scenarios are set up to simulate the total carbon emission of construction waste recycling under the current recycling rates. The carbon-emission-saving potential under S1 and S2 will be 51.43% and 102.86% in the year 2031 compared with the current scenario.

This study contributes to the body of knowledge in three aspects. Firstly, the proposed methods can also be applied to other regions, especially when the data of construction waste are insufficient. Secondly, this study not only estimated the carbon emission of recycling activities (environmental cost) but considered the equivalent amount of carbon emissions generated from alternative materials (environmental benefit), which is more in line with practice. The research outcomes would more clearly reveal the environmental benefits generated by construction waste recycling. Thirdly, this study estimated the total carbon emission of construction waste recycling in Shanghai from 2022 to 2031. Obtaining such data would help decision makers to set up more scientific management strategies and the action plan for Carbon Dioxide Peaking Before 2030 from the aspect of improving the recycling rate of construction waste and usage rate of recycled materials, which could further contribute to the achievement of China’s 2060 carbon neutral goal by focusing on one of the most important economic regions: Shanghai City. However, this study has three main limitations. The first limitation is the assumptions. For example, the production of mortar waste recycling is not ignored, thereby affecting the accuracy of the results. The second limitation is that only the construction waste generated from new construction activities are quantified in this research. If other types of waste, such as demolition waste, construction clay and mud, are considered, then the carbon emission will be higher. The third one is that this study does not compare the outcomes with other disposals, such as traditional landfill practice. If such comparisons can be done, then it could provide decision makers with a clearer understanding of the carbon-saving potential generated from recycling construction waste. Despite research limitations, the results can still provide regulatory authorities with theoretical methods and data to establish strategies and policies on promoting waste recycling and cutting down on CO_2_ emissions. 

## Figures and Tables

**Table 1:** The composition and current recycling rate of construction waste in Shanghai.

Construction Waste	Weight (kg)	Percentage	Recycling Rate	Reference
Concrete	429	42.9%	90%	Li [15]
Brick or block	383	38.3%	50%	Tang [31]
Wood	112	11.2%	50%	Tang [31]
Steel	65	6.5%	50%	Tang [31]
Mortar	11	1.1%	40%	Li [15]
Total	1000	100	-	-

**Table 2:** Value of construction area and construction waste in Shanghai from 2010 to 2021.

Year	Construction Area/10,000 m^2^	Construction Waste/10,000 t
2010	11,295.03	451.8012
2011	12,983.32	519.3328
2012	13,249.97	529.9988
2013	13,516.58	540.6632
2014	14,690.18	587.6072
2015	15,095.33	603.8132
2016	15,111.24	604.4496
2017	15,362.25	614.49
2018	14,672.37	586.8948
2019	14,802.97	592.1188
2020	15,001.66	600.0664
2021	15,210.68	608.4272

**Table 3:** Calculation results of the sequence B and $Y_{n}$.

Year	$x^{\left(0\right)}$	$x^{\left(1\right)}$	Sequence B		$Y_{n}$
2010	451.8012	451.8012	−711.4676	1	519.3328
2011	519.3328	971.134	−1236.1334	1	529.9988
2012	529.9988	1501.1328	−1771.4644	1	540.6632
2013	540.6632	2041.796	−2335.5996	1	587.6072
2014	587.6072	2629.4032	−2931.3098	1	603.8132
2015	603.8132	3233.2164	−3535.4412	1	604.4496
2016	604.4496	3837.666	−4144.911	1	614.49
2017	614.49	4452.156	−4745.6034	1	586.8948
2018	586.8948	5039.0508	−5335.1102	1	592.1188
2019	592.1188	5631.1696	−5931.2028	1	600.0664
2020	600.0664	6231.236	−6535.4496	1	608.4272
2021	608.4272	6839.6632	−711.4676	1	519.3328

**Table 4:** The average transportation distances of construction waste in Shanghai.

	Downtown *	Jiading	Baoshan	Qingpu	Songjiang	Jinshan	Fengxian	Minhang	Pudong	Congming
$L_{i}$ (km)	24.5	28.2	6.1	23.7	35.1	43.8	15.5	30.9	54.8	24.5
$S_{i}$	18.78%	4.55%	4.29%	4.63%	9.26%	3.69%	4.29%	14.75%	30.36%	5.40%
*D* (km)	35.29									

* Downtown includes Huangpu, Xuhui, Changning, Jingan, Putuo, Hongkou and Yangpu districts.

**Table 5:** Carbon emissions of key activities in this study (1 t).

Activities	Carbon Emission (kg CO_2_−e)	References
Transportation	0.228/kg-km	Ecoinvent [35]
Concrete and mortar recycling	0.017/kg	Ecoinvent [35]
Brick recycling	32.25/kg	Wang [36]
Steel production	2100/kg	Gu [23]
Wood production	39.95/kg	Wu, Duan [17]
Wood recycling	52.61/kg	Wu, Duan [17]
Diesel production	1462 mg/MJ	Yang [34]
Electricity production	317,000 mg/MJ	Yang [34]

**Table 6:** Carbon emissions of concrete, brick, steel, and mortar recycling (1 t).

Waste Composition	Activities	Carbon Emission (kg CO_2_−e)	Quantity (t)	Carbon Emission of 1 t Construction Waste (kg CO_2_−e)
Concrete recycling	Transportation (1.1)	8.05	0.429	3.62
	Recycling activities (2.1–2.3)	3.09		
	Recycling credits (2.4)	−2.71		
	Subtotal	8.43		
Brick recycling	Transportation (1.2)	8.05	0.383	13.72
	Recycling activities (3.1–3.3)	27.90		
	Recycling credits (3.4)	−0.13		
	Subtotal	35.82		
Steel recycling	Transportation (1.3)	11.42	0.065	−117.72
	Recycling activities (4.1)	911.26		
	Recycling credits (4.2)	−2733.77		
	Subtotal	−1811.09		
Mortar recycling	Transportation (1.4)	8.05	0.011	0.12
	Recycling activities (5.1)	3.09		
	Subtotal	11.14		
Wood recycling	Transportation (1.5)	11.42	0.112	−0.14
	Recycling activities (6.1)	39.95		
	Recycling credits (6.2)	−52.61		
	Subtotal	−1.24		
Total				−100.4

**Table 7:** Sensitivity analysis of Carbon emissions of waste recycling (1 t).

Sensitivity Factor	Waste Composition	Rate of Change (under −10%)	Rate of Change (under 10%)
Transportation distance	Carbon emission of concrete recycling	−9.55%	9.55%
	Carbon emission of brick recycling	−2.25%	2.25%
	Carbon emission of wood recycling	9.21%	−9.21%
	Carbon emission of steel recycling	0.06%	−0.06%
	Carbon emission of mortar recycling	−7.23%	7.23%
Recycling rate	Carbon emission of concrete recycling	−3.67%	3.67%
	Carbon emission of brick recycling	−7.79%	7.79%
	Carbon emission of wood recycling	3.22%	−3.22%
	Carbon emission of steel recycling	5.03%	−5.03%
	Carbon emission of mortar recycling	−2.77%	2.77%

## Data Availability

The data presented in this study are available on request from the corresponding author.
